# P-1201. Treading New Paths: Dalbavancin in Gram-positive Diabetic Foot Infections

**DOI:** 10.1093/ofid/ofaf695.1394

**Published:** 2026-01-11

**Authors:** Baiju Patel, Shiwei Zhou, Hojin Lee, Adam M Ressler, Brian M Schmidt

**Affiliations:** University of Michigan, Ann Arbor, MI; Michigan Medicine, Ann Arbor, Michigan; University of Michigan, Michigan Medicine, Canton, Michigan; University of Michigan, Ann Arbor, MI; University of Michigan, Ann Arbor, MI

## Abstract

**Background:**

Dalbavancin is a long-acting antibiotic approved for the treatment of skin and soft tissue infection. There is limited data on the effectiveness of dalbavancin compared to standard-of-care (SoC) treatment in patients with diabetic foot osteomyelitis (DFO).

**Figure:**
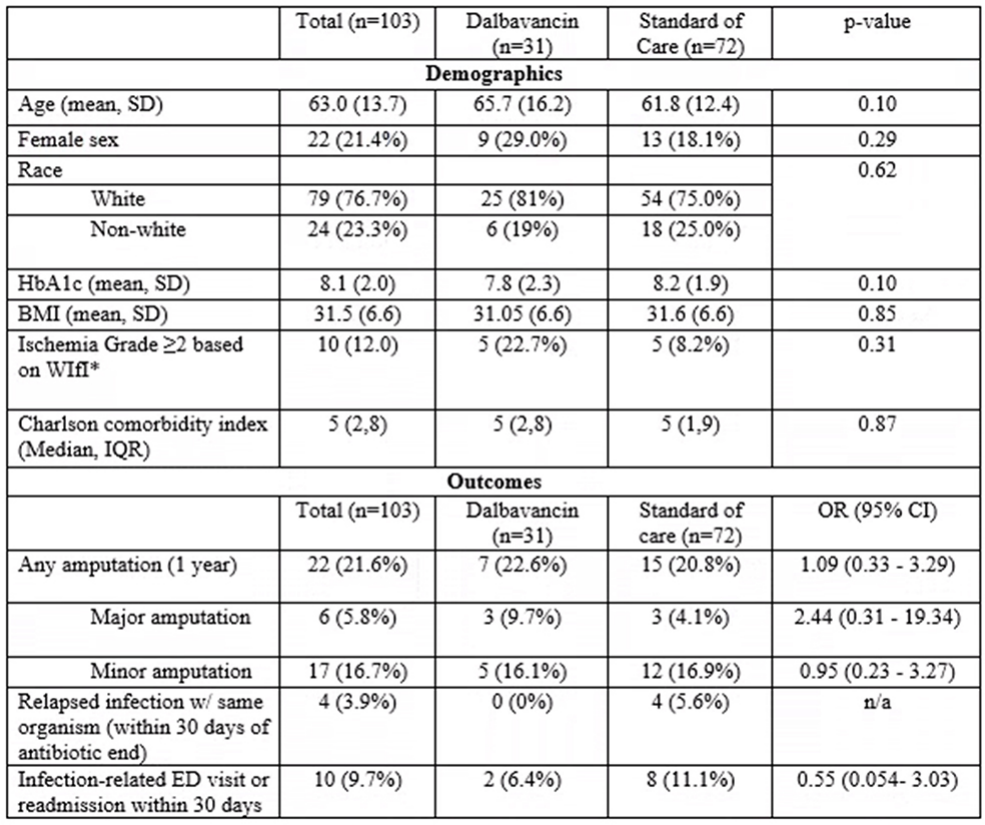
Demographics and outcomes of dalbavancin group compared to standard of care group *Ischemia grade from the WIfI (wound, ischemia, foot infection) classification system. Grade 0: ABI >0.8 or Toe pressure >60 mmHg; Grade 1: ABI 0.6-0.79 or Toe pressure 40-59; Grade 2: ABI 0.4-0.59 or Toe pressure 30-39; Grade 3: ABI < 0.40 or Toe pressure < 30

**Figure 1:**
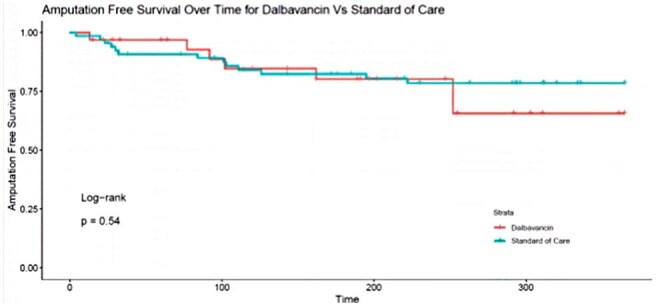
Kaplan-Meier survival analysis for freedom from any amputation as a function of treatment with dalbavancin vs standard of care.

**Methods:**

A retrospective observational cohort analysis was done for adult patients at Michigan Medicine diagnosed with DFO with ≥1 gram positive organism present on tissue culture from Jan 2022 - Dec 2024 who were treated with dalbavancin or SoC oral antibiotics with up to 1 year follow up. The primary outcome was freedom from major (above/below knee) and minor (toe/transmetatarsal) amputation at 1 year. Secondary outcomes included treatment failure, defined as relapse of infection with the same organism at the same location within 30 days of completing antibiotics, and infection-related readmission within 30 days.

**Results:**

Of 103 patients included, 31 were treated with dalbavancin and 72 with SoC (primarily linezolid). Mean age of the cohort was 63; 21% were female, 77% were white, with a median Charlson comorbidity index of 5 and mean HbA1c of 8.1. The dalbavancin group had numerically more patients with severe peripheral arterial disease but this was not statistically significant. Otherwise, the two groups were similar with regards to measured demographics and comorbid conditions (Table).

Amputation rates 1 year following diagnosis were similar between the dalbavancin and SoC groups (22.6% vs 20.8%, OR 1.09, CI 0.33-3.29, Figure). Major amputation was higher in the dalbavancin group compared to the SoC group but did not reach statistical significance (9.7% vs 4.1%, OR 2.44, CI 0.31-19.34). Rates of minor amputation were similar among the two groups (16.1% vs 16.9%, OR 0.95, CI 0.23 - 3.27). The dalbavancin group had no recurrent infections and lower rates of infection-related ED visits or readmissions within 30 days compared to SoC (6.4% vs 11.1%, OR 0.55, CI 0.054- 3.03).

**Conclusion:**

In this cohort of patients with DFO, dalbavancin treatment had similar rates of amputation at 1 year compared to SoC. The dalbavancin group trended toward lower rates of infection relapse and infection related readmission. This study suggests that dalbavancin may be an effective therapeutic option in DFO treatment.

**Disclosures:**

All Authors: No reported disclosures

